# Supplementary material for: T Cells Directed against the Metastatic Driver Chondromodulin-1 in Ewing Sarcoma: Comparative Engineering with CRISPR/Cas9 vs. Retroviral Gene Transfer for Adoptive Transfer
Source: Cancers (Basel). 2022 Nov 8;14(22):5485. doi: 10.3390/cancers14225485 (PMC9688113; doi:10.3390/cancers14225485)
Supplement: Supplementary file 1 [file cancers-14-05485-s001.zip › Supplementary figures 2nd submission.pptx]

## Slide 1
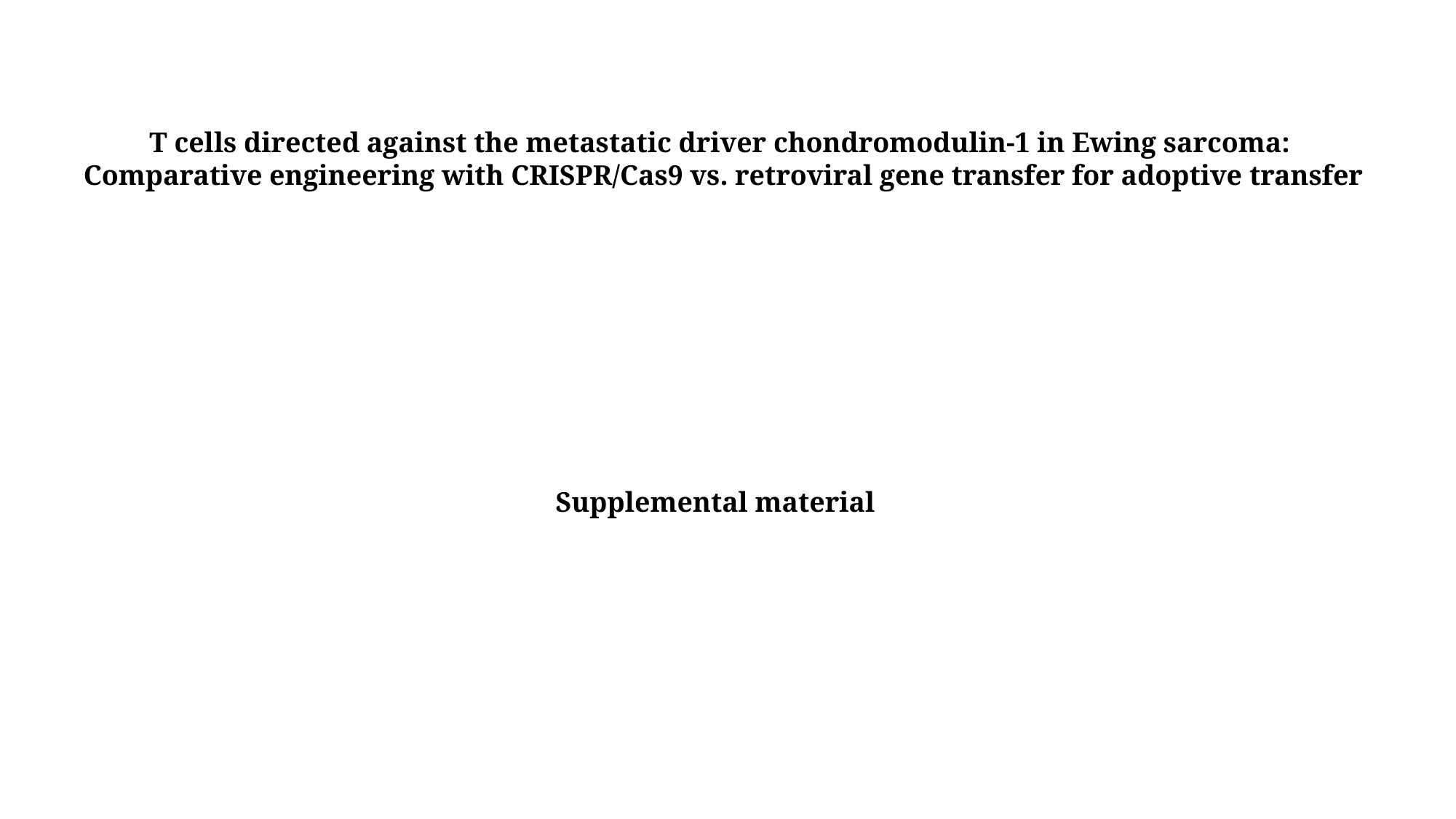

T cells directed against the metastatic driver chondromodulin-1 in Ewing sarcoma:
 Comparative engineering with CRISPR/Cas9 vs. retroviral gene transfer for adoptive transfer
Supplemental material

## Slide 2
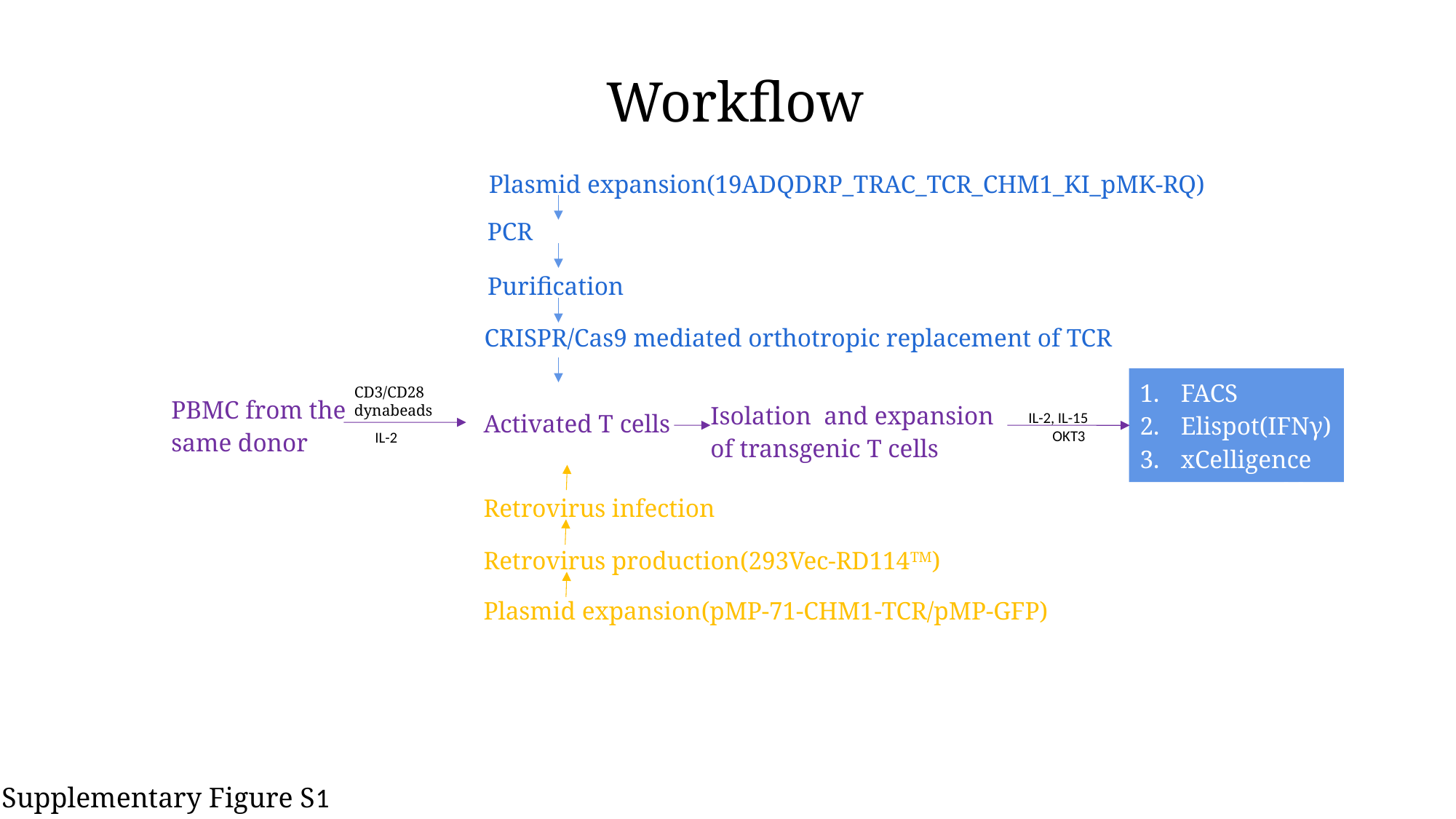

Workflow
Plasmid expansion(19ADQDRP_TRAC_TCR_CHM1_KI_pMK-RQ)
PCR
Purification
CRISPR/Cas9 mediated orthotropic replacement of TCR
FACS
Elispot(IFNγ)
xCelligence
Western blot
CD3/CD28 dynabeads
PBMC from the same donor
Isolation and expansion of transgenic T cells
IL-2, IL-15
 OKT3
Activated T cells
 IL-2
Retrovirus infection
Retrovirus production(293Vec-RD114TM)
Plasmid expansion(pMP-71-CHM1-TCR/pMP-GFP)
Supplementary Figure S1

## Slide 3
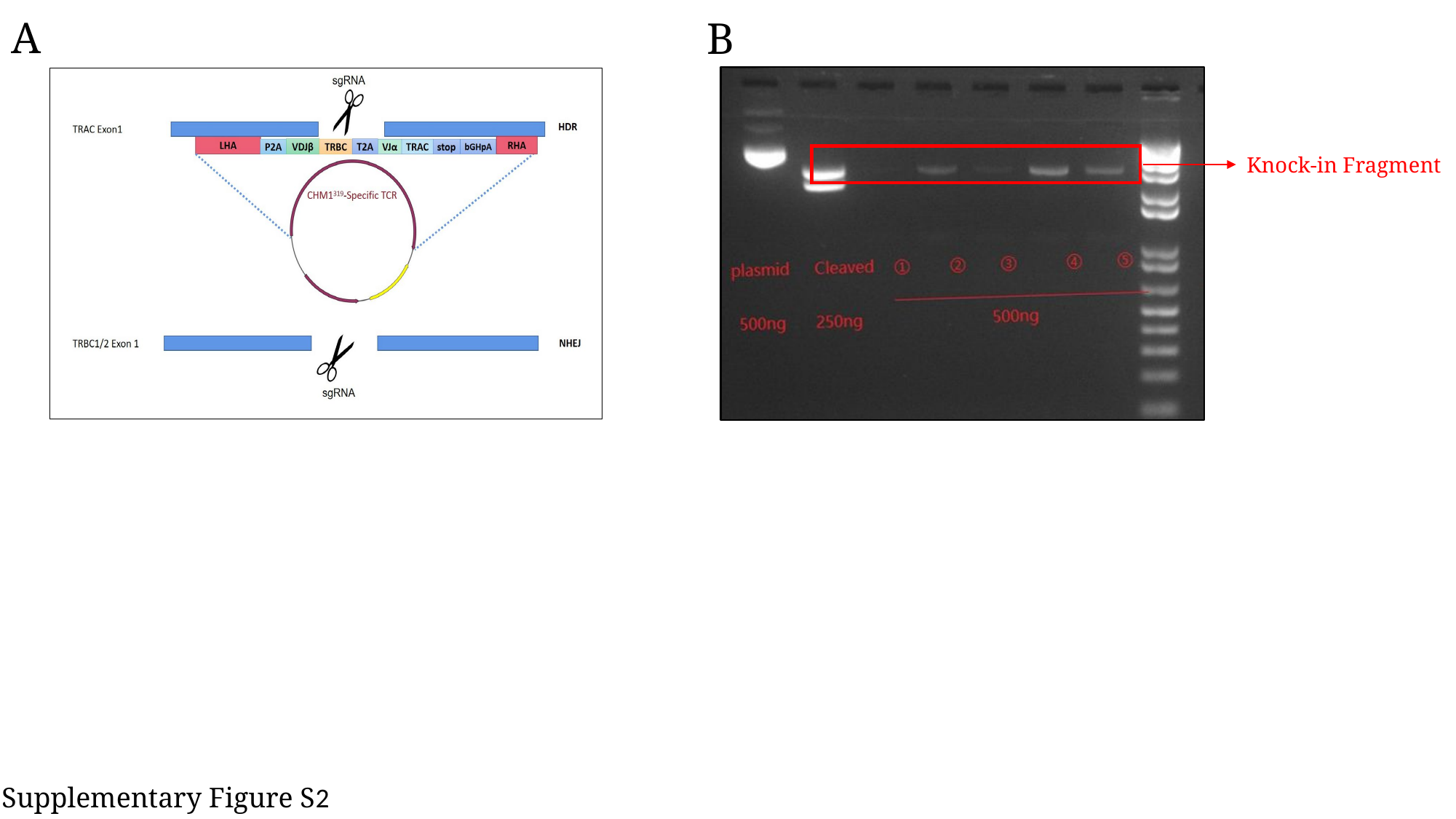

A
B
Knock-in Fragment
Supplementary Figure S2

## Slide 4
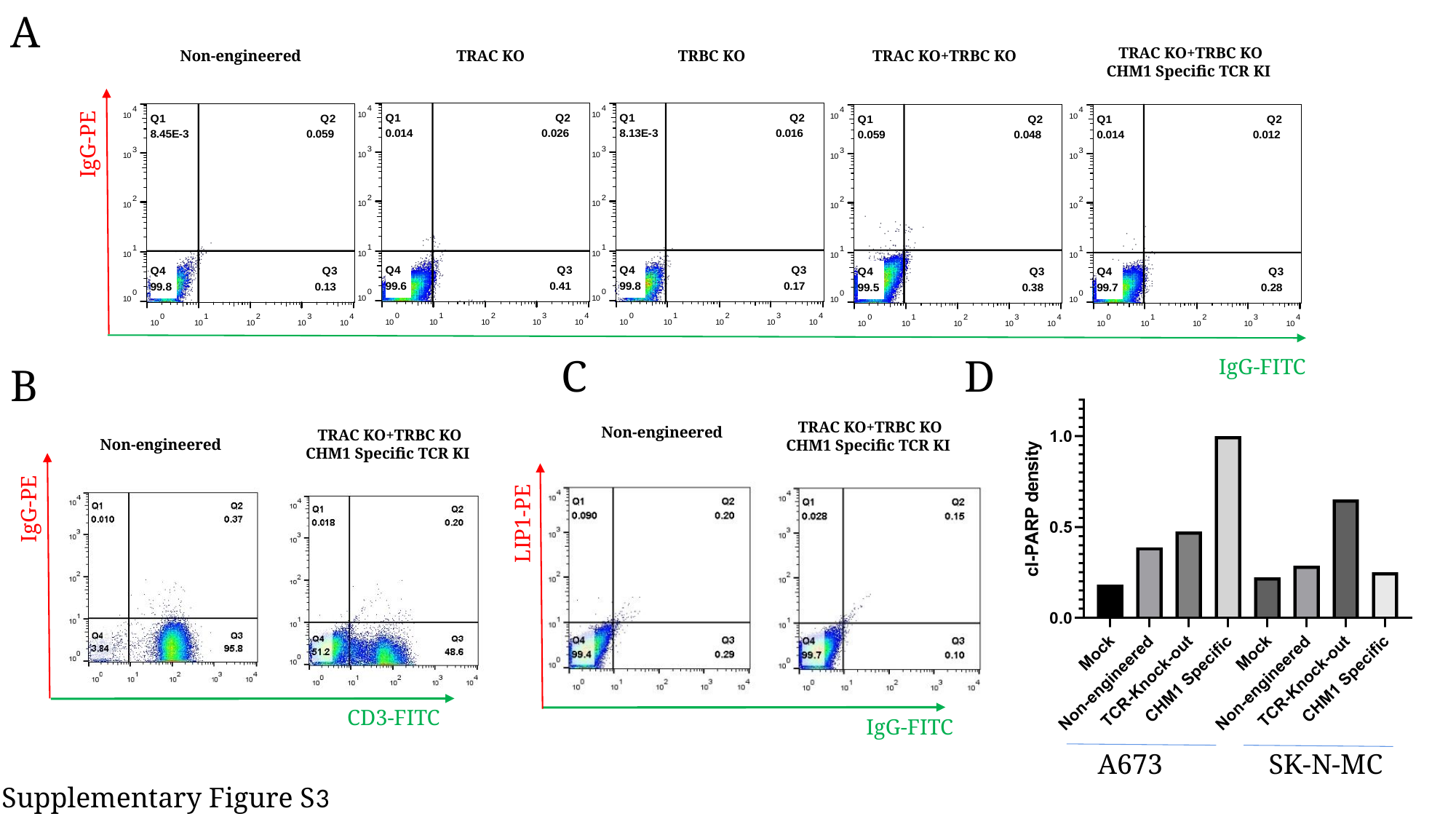

A
 TRAC KO+TRBC KO
 CHM1 Specific TCR KI
Non-engineered
TRAC KO
TRBC KO
TRAC KO+TRBC KO
IgG-PE
C
D
IgG-FITC
B
 TRAC KO+TRBC KO
 CHM1 Specific TCR KI
Non-engineered
 TRAC KO+TRBC KO
 CHM1 Specific TCR KI
Non-engineered
IgG-PE
LIP1-PE
CD3-FITC
IgG-FITC
A673
SK-N-MC
Supplementary Figure S3

## Slide 5
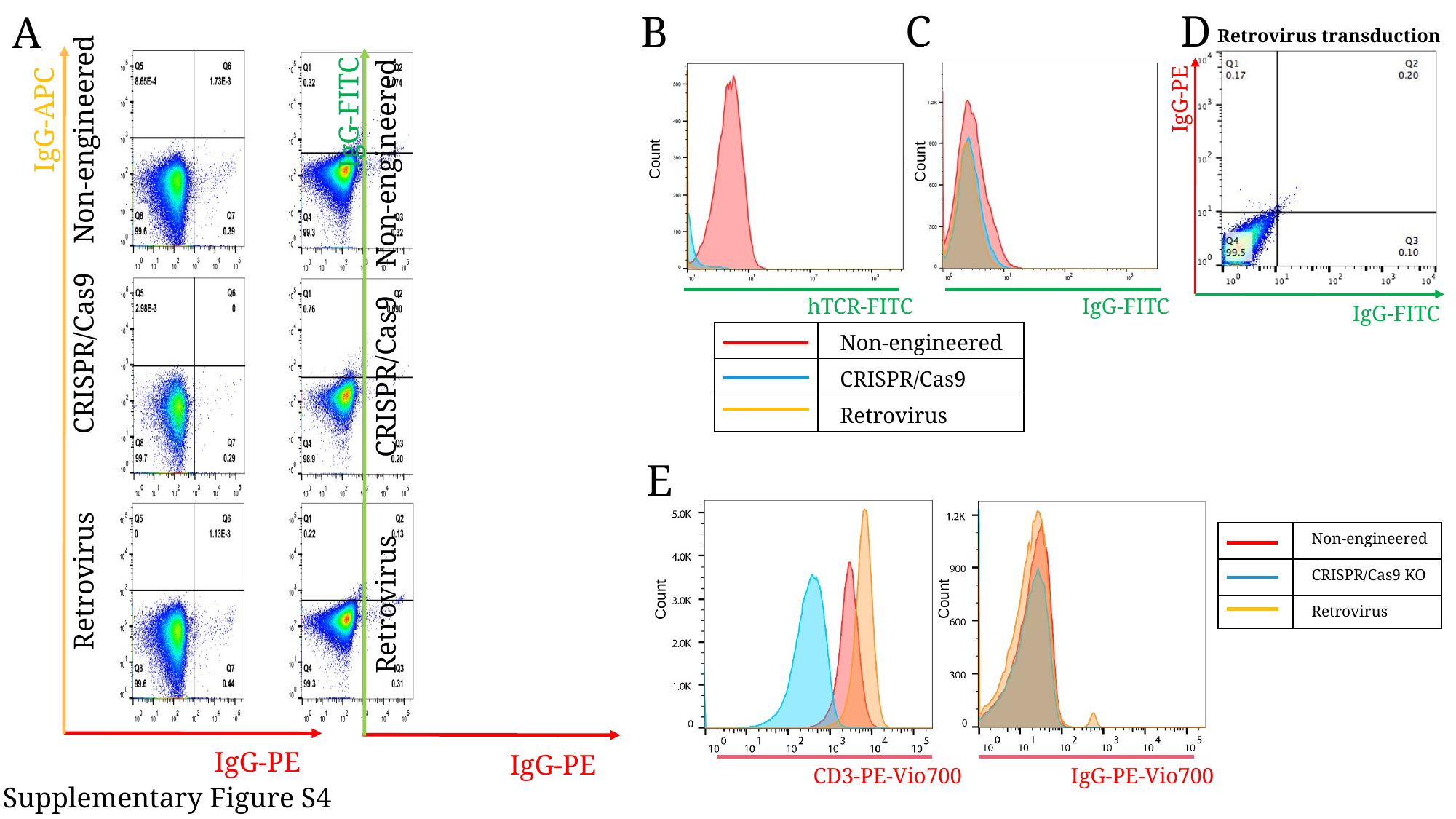

C
D
B
A
Retrovirus transduction
IgG-PE
IgG-FITC
IgG-APC
Count
Count
 hTCR-FITC IgG-FITC
IgG-FITC
Retrovirus CRISPR/Cas9 Non-engineered
| | Non-engineered |
| --- | --- |
| | CRISPR/Cas9 |
| | Retrovirus |
Retrovirus CRISPR/Cas9 Non-engineered
E
| | Non-engineered |
| --- | --- |
| | CRISPR/Cas9 KO |
| | Retrovirus |
Count
Count
IgG-PE
IgG-PE
 CD3-PE-Vio700 IgG-PE-Vio700
Supplementary Figure S4

## Slide 6
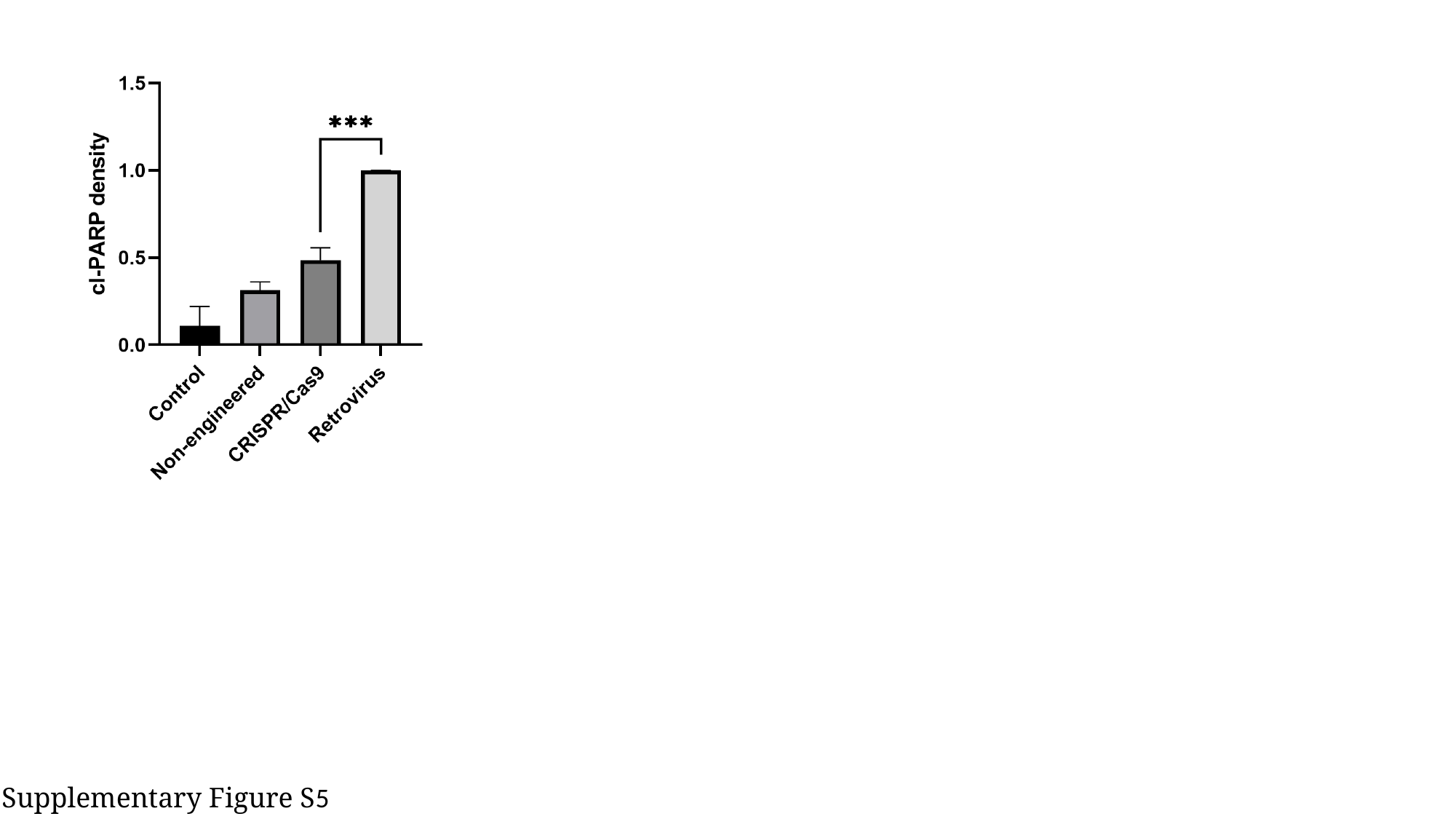

Supplementary Figure S5

## Slide 7
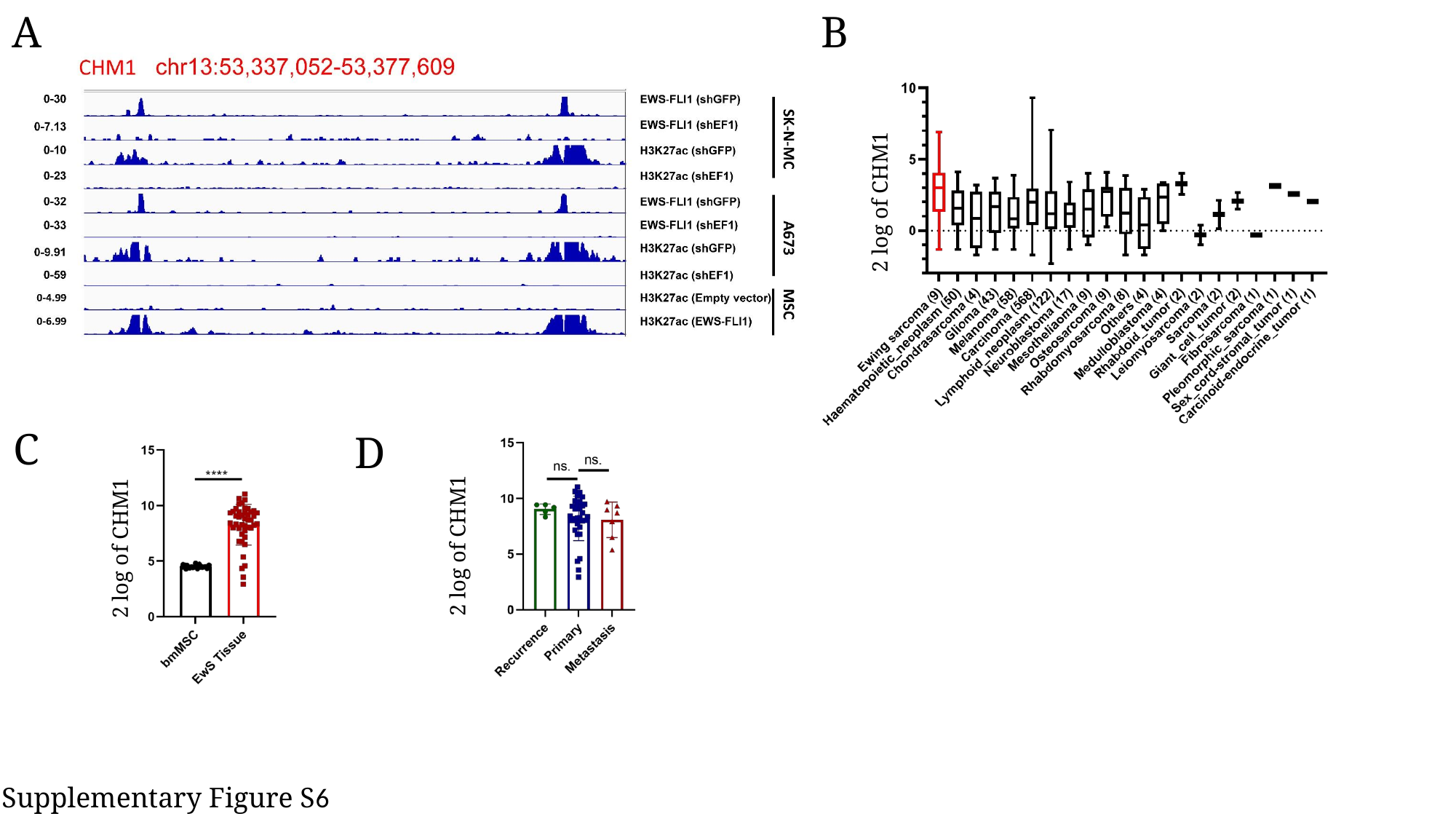

A
B
2 log of CHM1
C
D
2 log of CHM1
2 log of CHM1
Supplementary Figure S6

## Slide 8
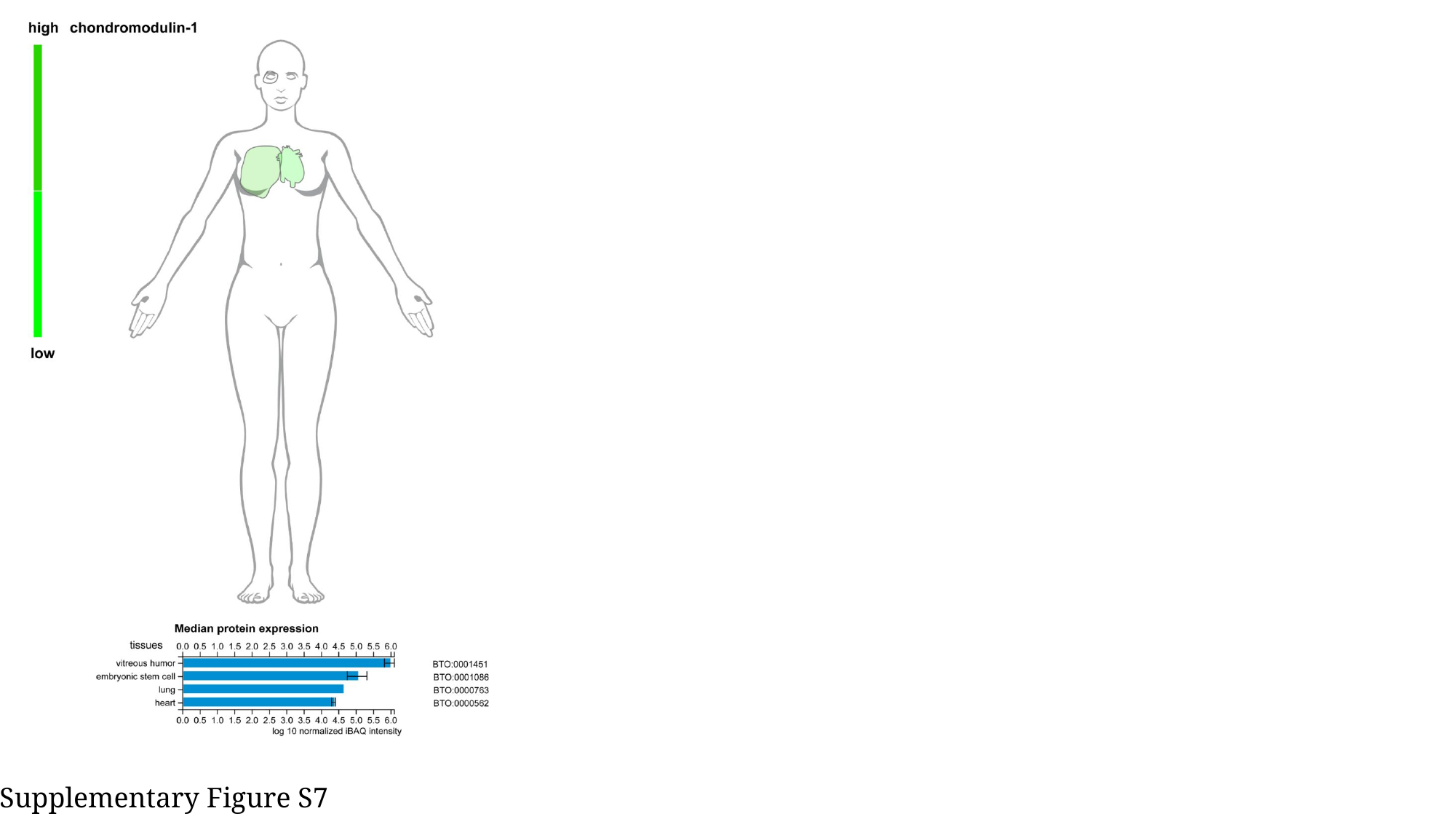

Supplementary Figure S7
