# Supplementary material for: T Cells Directed against the Metastatic Driver Chondromodulin-1 in Ewing Sarcoma: Comparative Engineering with CRISPR/Cas9 vs. Retroviral Gene Transfer for Adoptive Transfer
Source: Cancers (Basel). 2022 Nov 8;14(22):5485. doi: 10.3390/cancers14225485 (PMC9688113; doi:10.3390/cancers14225485)
Supplement: Supplementary file 1 [file cancers-14-05485-s001.zip › Supplementary table Xue et al. Cancers.pdf]

SUPPLEMENTARY **TABLE S1:** DNA SEQUENCE OF KNOCK-IN CHM1^{319}-TCR

LHA	CTGCCTTTACTCTGCCAGAGTTATATTGCTGGGGTTTTGAAGAAGATCCTATTAAATAAAAGAATAAGCAGTATTATTAAGTAGCCCTGCATTTCAGGTTTCCTTGAGTGGCAGGCCAGGCCTGGCCGTGAACGTTCACTGAAATCATGGCCTCTTGGCCAAGATTGATAGCTTGTGCCTGTCCCTGAGTCCCAGTCCATCACGAGCAGCTGGTTTCTAAGATGCTATTTCCCGTATAAAGCATGAGACCGTGACTTGCCAGCCCCACAGAGCCCCGCCCTTGTCCATCACTGGCATCTGGACTCCAGCCTGGGTTGGGGCAAAGAGGGAAATGAGATCATGTCCTAACCCTGATCCTCTTGTCCCACAGATATCCAGAACCCTGACCCTGCCGTG
P2A	GGCAGCGGCGCCACCAACTTCAGCCTGCTGAAGCAGGCCGGCGACGTGGAAGAGAACCCCGGGCCC
VDJβ	ATGCTGTCTCCAGATCTGCCTGACAGCGCCTGGAACACCCGGCTGCTGTGCAGAGTGATGCTGTGCCTGCTGGGAGCCGGATCTGTGGCTGCTGGCGTGATCCAGAGCCCCAGACACCTGATCAAAGAGAAGAGAGAGACAGCCACCCTGAAGTGCTACCCCATCCCCAGGCACGACACCGTGTACTGGTATCAGCAGGGCCCAGGCCAGGACCCCCAGTTCCTGATCAGCTTCTACGAGAAGATGCAGAGCGAC
TRBC with additional cysteine bridges	AAGGGCAGCATCCCCGACAGATTCAGCGCCCAGCAGTTCAGCGACTACCACAGCGAGCTGAACATGAGCAGCCTGGAACTGGGCGACAGCGCCCTGTACTTCTGCGCCTCTAGCTTCCTGGGCGAGAAAACCGAGGCATTCTTTGGGCAGGGCACCAGACTGACCGTGGTGGAGGATCTGAGAAATGTGACTCCACCCAAGGTCTCCTTGTTTGAGCCATCAAAAGCAGAGATTGCAAACAAACAAAAGGCTACCCTCGTGTGCTTGGCCAGGGGCTTCTTCCCTGACCACGTGGAGCTGAGCTGGTGGGTGAATGGCAAGGAGGTCCACAGTGGGGTCTGCACGGACCCTCAGGCCTACAAGGAGAGCAATTATAGCTACTGCCTGAGCAGCCGCCTGAGGGTCTCTGCTACCTTCTGGCACAATCCTCGAAACCACTTCCGCTGCCAAGTGCAGTTCCATGGGCTTTCAGAGGAGGACAAGTGGCCAGAGGGCTCACCCAAACCTGTCACACAGAACATCAGTGCAGAGGCCTGGGGCCGAGCAGACTGTGGAATCACTTCAGCATCCTATCATCAGGGGGTTCTGTCTGCAACCATCCTCTATGAGATCCTACTGGGGAAGGCCACCCTATATGCTGTGCTGGTCAGTGGCCTGGTGCTGATGGCCATGGTCAAGAAAAAAAATTCC
T2A	GGCAGCGGCGAGGGCAGAGGAAGTCTGCTAACATGCGGTGACGTCGAGGAGAATCCTGGACCT
VJα	ATGACCAGCATCCGGGCCGTGTTCATCTTCCTGTGGCTGCAGCTGGACCTCGTGAACGGCGAGAACGTGGAACAGCACCCCAGCACCCTGAGCGTGCAGGAAGGCGATAGCGCCGTGATCAAGTGCACCTACAGCGACTCCGCCAGCAACTACTTCCCCTGGTACAAGCAGGAACTGGGAAAGCGGCCCCAGCTGATCATCGACATCCGGTCCAACGTGGGAGAGAAGAAGGACCAGCGGATCGCCGTGACCCTG
TRAC with additional cysteine bridges	AACAAGACCGCCAAGCACTTCTCCCTGCACATCACCGAGACACAGCCCGAGGACTCCGCCGTGTACTTTTGTGCCGCTTCTGCCGGCGGATCCCAGGGCAATCTGATCTTCGGCAAGGGCACCAAGCTGAGCGTGAAGCCCAACATCCAGAACCCAGAACCTGCTGTGTACCAGTTAAAAGATCCTCGGTCTCAGGACAGCACCCTCTGCCTGTTCACCGACTTTGACTCCCAAATCAATGTGCCGAAAACCATGGAATCTGGAACGTTCATCACTGACAAATGCGTGCTGGACATGAAAGCTATGGATTCCAAGAGCAATGGGGCCATTGCCTGGAGCAACCAGACAAGCTTCACCTGCCAAGATATCTTCAAAGAGACCAACGCCACCTACCCCAGTTCAGACGTTCCCTGTGATGCCACGTTGACTGAGAAAAGCTTTGAAACAGATATGAACCTAAACTTTCAAAACCTGTCAGTTATGGGACTCCGAATCCTCCTGCTGAAAGTAGCCGGATTTAACCTGCTCATGACGCTGAGGCTGTGGTCCAGT
Stop	TGA

Poly A	CTAGAGCTCGCTGATCAGCCTCGACTGTGCCTTCTAGTTGCCAGCCATCTG<br>TTGTTTGCCCCTCCCCCGTGCCTTCCTTGACCCTGGAAGGTGCCACTCCCA<br>CTGTCCTTTCCTAATAAAATGAGGAAATTGCATCGCATTGTCTGAGTAGGTG<br>TCATTCTATTCTGGGGGGTGGGGTGGGGCAGGACAGCAAGGGGGAGGAT<br>TGGGAAGAGAATAGCAGGCATGCTGGGGA
RHA	TACCAGCTGAGAGACTCTAAATCCAGTGACAAGTCTGTCTGCCTATTCACC<br>GATTTTGATTCTCAAACAAATGTGTCACAAAGTAAGGATTCTGATGTGTATA<br>TCACAGACAAAACTGTGCTAGACATGAGGTCTATGGACTTCAAGAGCAAC<br>AGTGCTGTGGCCTGGAGCAACAAATCTGACTTTGCATGTGCAAACGCCTTC<br>AACAACAGCATTATTCCAGAAGACACCTTCTTCCCCAGCCCAGGTAAGGG<br>CAGCTTTGGTGCCTTCGCAGGCTGTTTCCTTGCTTCAGGAATGGCCAGGTT<br>CTGCCCAGAGCTCTGGTCAATGATG
